# Trends of pH decrease in the Mediterranean Sea through high frequency observational data: indication of ocean acidification in the basin

**DOI:** 10.1038/srep16770

**Published:** 2015-11-26

**Authors:** Susana Flecha, Fiz F. Pérez, Jesús García-Lafuente, Simone Sammartino, Aida. F. Ríos, I. Emma Huertas

**Affiliations:** 1 Instituto de Ciencias Marinas de Andalucía, (CSIC), Polígono Río San Pedro, s/n, 11519, Puerto Real, Cádiz, Spain; 2 Instituto de Investigaciones Marinas, (CSIC), Eduardo Cabello, 6, 36208, Vigo, Spain; 3 Physical Oceanography Group, University of Málaga, Campus de Teatinos s/n, 29071, Málaga, Spain

## Abstract

A significant fraction of anthropogenic carbon dioxide (CO_2_) released to the atmosphere is absorbed by the oceans, leading to a range of chemical changes and causing ocean acidification (OA). Assessing the impact of OA on marine ecosystems requires the accurate detection of the rate of seawater pH change. This work reports the results of nearly 3 years of continuous pH measurements in the Mediterranean Sea at the Strait of Gibraltar GIFT time series station. We document a remarkable decreasing annual trend of −0.0044 ± 0.00006 in the Mediterranean pH, which can be interpreted as an indicator of acidification in the basin based on high frequency records. Modeling pH data of the Mediterranean outflow allowed to discriminate between the pH values of its two main constituent water masses, the Levantine Intermediate Water (LIW) and the Western Mediterranean Deep Water (WMDW). Both water masses also exhibited a decline in pH with time, particularly the WMDW, which can be related to their different biogeochemical nature and processes occurring during transit time from formation sites to the Strait of Gibraltar.

CO_2_ emissions from fossil fuels burning and land use change since the industrial revolution have caused a considerable increase in atmospheric CO_2_ concentrations^1^. Recent investigations have estimated that cumulative emissions of CO_2_ have reached in the period from 1870 to 2013 about 535 ± 55 GtC^2^. However, a significant CO_2_ amount has been captured from the atmosphere by natural sinks, such as the terrestrial biosphere and the ocean. In particular, the global oceans have absorbed about 30% of the anthropogenic carbon emissions over the past 200 years^3^.

The withdrawal of CO_2_ by the oceans has, however, drastic consequences for the marine environment, as it originates a rise in average surface ocean concentration of H^+^ that leads to a pH decrease in seawater and a range of chemical changes known collectively as “the other CO_2_ problem” or the ocean acidification (OA) phenomenon^4^,^5^. The impact of OA on marine biogeochemical cycles and biota has been well documented by laboratory studies and already observed to occur in certain ocean areas^6^,^7^,^8^,^9^. It has been suggested that the Mediterranean Sea (MS) represents one of the world’s most sensitive ocean regions to increasing atmospheric CO_2_ and the subsequent OA^10^,^11^. Nevertheless, contradicting observations of pH changes with time have been reported in the basin. A recent study affirms that the MS is already acidified, although distinct OA rates are provided depending on the degree of anthropogenic carbon accumulation by a particular sub-basin, with regional pH decreases oscillating between −0.055 to −0.156 pH units with respect to the preindustrial levels^12^. On the other hand, another work points out to pH reductions between 0.005 to 0.06 pH units due to the anthropogenic carbon storage in the basin during the same period^13^. Moreover, it has been proposed that the pH decline would be amplified in the MS due to its higher capacity for CO_2_ absorption in relation to open ocean regions^14^,^15^ and the relatively short ventilation times of its water masses^14^, statements that were challenged recently by a modelling approach^13^ which indicates that the average anthropogenic change in surface pH does not differ significantly from the global-ocean average.

Considerable efforts have been made over the last decade to characterize the carbonate system in Mediterranean water masses^16^,^17^,^18^, to explore how much anthropogenic CO_2_ has been taken up by this semi-enclosed sea and to assess the corresponding pH diminution^13^,^14^,^19^,^20^. Most of these studies rely on discrete data sets that extend over a specific period of time or cover a particular Mediterranean sub-region. Thus, sometimes, these studies lead to discrepant conclusions when comparisons between data acquired in different periods, using distinct techniques and/or in distant locations are made.

The assessment of the marine ecosystems responses to the oceanic CO_2_ uptake requires then sustained observations that provide the needed high frequency data about changes in ocean chemistry. At present, this type of measurements is being collected in a few ocean time-series^21^. The continuous monitoring of the carbon system parameters (and other tracers for hydrography and biochemistry) in these key sites have supplied relevant information on the ocean CO_2_ sink and the derived pH changes in different regions, confirming a general OA trend over the past two decades.

Sustained time-series observations also started in the Strait of Gibraltar (SG) a decade ago through the establishment of the GIFT (Gibraltar Fixed Time Series) observatory, since this region represents a privileged site to observe the evolution of the Mediterranean waters over time. This narrow channel (14 km wide in its narrowest section) is the only connection of the MS with the North Atlantic, thereby playing a major role in the global circulation and biogeochemistry of the basin^22^,^23^.

The circulation pattern in the SG has been traditionally described as a two-layer system, with surface Atlantic water (AW) flowing eastwards to the MS and the Mediterranean Outflow Water (MOW) moving westward to the Atlantic Ocean underneath. The AW enters the MS and flows clockwise in the Alboran Sea (AS, Fig. S1). Subsequent surface circulation patterns at the basin level are influenced by deep and intermediate water formation driven by strong winds, which is in turn affected and amplified by topography. Deep and intermediate waters are formed in four major areas: the Levantine Basin (LB, Fig. S1), the source of the Levantine Intermediate Water (LIW); the Gulf of Lions (GL, Fig. S1) where the Western Mediterranean Deep Water (WMDW) is formed; and two adjacent regions, the Adriatic and the Aegean Seas (AdS, AeS, respectively in Fig. S1), which together merge to form the Eastern Mediterranean Deep Water (EMDW). The MOW that leaves the basin through the Strait of Gibraltar is then a mixture of these intermediate and deep waters, fundamentally LIW, which flows across the Strait of Sicily (SS, Fig. S1) into the Western Mediterranean basin, and the WMDW, which occupies the bottom layer^24^. The contribution of the AW that penetrates into the MS in surface to the final outflow exiting the basin is negligible^25^. By monitoring the hydrography and biogeochemistry of the MOW in the SG, the history and evolution of the main intermediate and deep Mediterranean water masses can be examined.

The exchange of waters of different ages carrying diverse concentrations of biogeochemical properties in the SG also influences global inventories in the two neighbour regions^26^,^27^. Regarding the marine carbon cycle, a net transport of anthropogenic carbon from the Atlantic towards the Mediterranean has been identified^19^,^20^,^26^, which has been indeed responsible for 25% of the basin-wide CO_2_ uptake over the last 200 years^13^.

In this work, we use pH measurements taken at the GIFT at a high sampling rate to assess temporal trends of pH change in Mediterranean waters. Data were obtained by autonomous sensors installed in a mooring line deployed at the Espartel Sill (ES, Fig. S1), which has been proven to be the most suitable section in the SG for monitoring the MOW^20^,^24^,^25^,^27^,^28^,^29^. Results presented here correspond to the first continuous pH records at a high temporal resolution registered in the channel from August 2012 to June 2015. Our work provides the first rates of pH decrease in the MOW and in its forming water masses separately, which can be considered indicators of OA in the basin, confirming previous evidence^12^,^13^,^15^. In addition, by using a simple model, we present a tool for tracking pH and its temporal variability in the MS.

## Results and Discussion

### Water masses and pH

**Figure 1:**
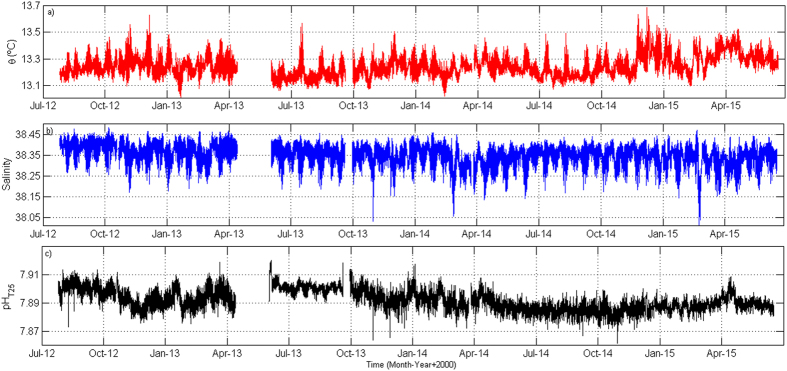
(**a**) Potential temperature (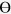), (**b**) Salinity obtained with the CT and (**c**) SAMI-pH data from August 2012 to June 2015.

From August 2012 to June 2015, the data collected at the mooring site fluctuated within small ranges of values. Potential temperature (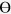) oscillated from 13.01 to 13.63 °C, salinity from 38.01 to 38.48 and pH in total scale at a reference temperature of 25 °C (pH_T25_) from 7.8618 to 7.9370 (Fig. 1). The pH_T25_ mean value was 7.8934 ± 0.0076 (*n *= 15937), which is in good agreement with the average pH in the MOW obtained from sustained spectrophotometric pH_T25_ measurements taken periodically within this layer at the GIFT stations from 2005 to 2014 (*n *= 102) and equivalent to 7.8875 ± 0.0124.

**Figure 2:**
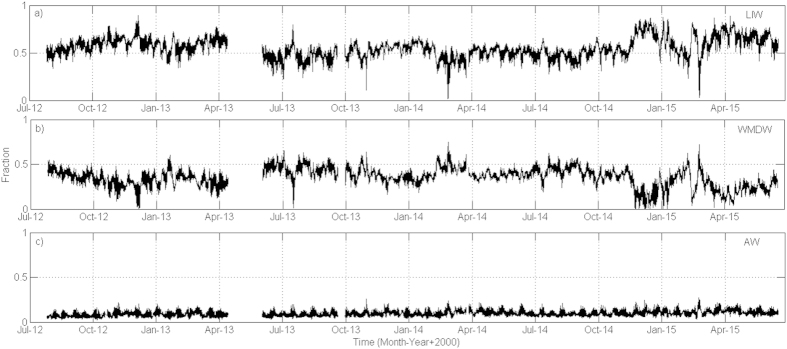
Fractions of the water masses forming the MOW during the monitoring period, according to the OMP analysis (see text): (**a**) LIW, (**b**) WDMW and (**c**) AW.

Water masses fractions variability in the MOW, discriminated with an OMP (Optimum MultiParameter) analysis^30^, (see *SI text* for more details), clearly describes seasonal and interannual fluctuations (Fig. 2). During the monitoring period, the LIW appeared to dominate the outflow most of the time, with a mean fraction of 0.55 ± 0.1 (Fig. 2a) whereas the WMDW showed a fraction of 0.36 ± 0.1 (Fig. 2b). Because of the sampling depth, the presence of the AW (Fig. 2c) was almost negligible within the MOW, with an average fraction of 0.09 ± 0.03, confirming historic observations^25^.

As previously described^24^,^31^, WMDW ventilation through the SG is modulated by several physical processes among which the WMDW formation events in the Gulf of Lions and the intensification of the Western Alboran Gyre (WAG, red arrow in Fig. S1) in the Alboran Sea are probably the most relevant. The WAG provides additional energy necessary to uplift deep waters from the Alboran Sea, facilitating the arrival of WMDW to the eastern entrance of the SG^31^. On the contrary, the absence or the relaxation of the WAG propitiates a major drainage of the LIW. Both processes can be traced in the monitoring station by gradual drops of potential temperature to values below 13.1°C^24^. During the monitoring period, such 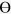 fall was clearly registered in January 2013 (Fig. 1a), which was accompanied by a concomitant rise in the WMDW fraction and a decrease in the LIW fraction (Fig. 2a,b). A similar pattern was also observed in February 2014 and February 2015 (Figs 1a and 2a,b). In a previous study that also used potential temperature as the parameter to trace water masses in the area^24^, WDMW formation events left a noticeable signature around March. Following the procedure described by these authors, the densest sample recorded in every semidiurnal tidal cycle was then extracted from our database to obtain a new subseries with semidiurnal sampling interval (not shown). In this new subseries, the major 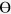 diminutions were found to commence by the middle of January 2013 and beginning of February 2014, with a gradual drop from ~13.2°C to values below 13.05 °C taking place in around 9 days in year 2013 and 13 days in 2014. The original temperature was recovered a month later approximately, a fact that may be partially attributed to the relaxation of the WAG.

### Modelled data

**Figure 3:**
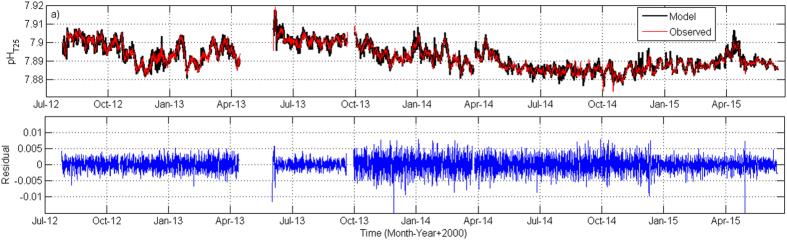
(**a**) pH_T25_ obtained with the SAMI device averaged to 84 h (red line) and modelled pH_T25_ (black line), (**b**) Residuals between observed values and modelled outputs.

As shown in Fig. 3a, the pH data modelled by a MLR (MultiLinear Regression, see *SI text*) faithfully reproduced *in situ* values (averaged to 84 h for comparison), and discrepancies between observational data and modelled outputs were in the order of ±0.005 pH units. Averaging such discrepancies to a 6 h period (Fig. 3b), residuals followed the tidal cycle pattern, thereby confirming that tidal variability was excluded in our estimations. However, higher residuals could still be detected during some periods. For instance, a sharp decline was evident in June 2013 (Fig. 3b), when pH data were recalculated from A_T_ and *p*CO_2_ measurements. This signal coincides with a rise in the pH_T25_ values (Fig. 1c) and it could be then attributed to the SAMI-CO_2_ instrument stabilization time. A second period of higher residual values was observed in October 2013 lasting until April 2014 (Fig. 3b), which may be attributable in this case to the change in the SAMI-pH sampling interval from hourly to bi-hourly. *p*CO_2_ data measured uninterruptedly in the MOW from June 2013 to December 2014 by a SAMI-CO_2_ device were also modelled by the MLR (see Fig. S3), with very low residuals being obtained, which supports the model robustness.

**Figure 4:**
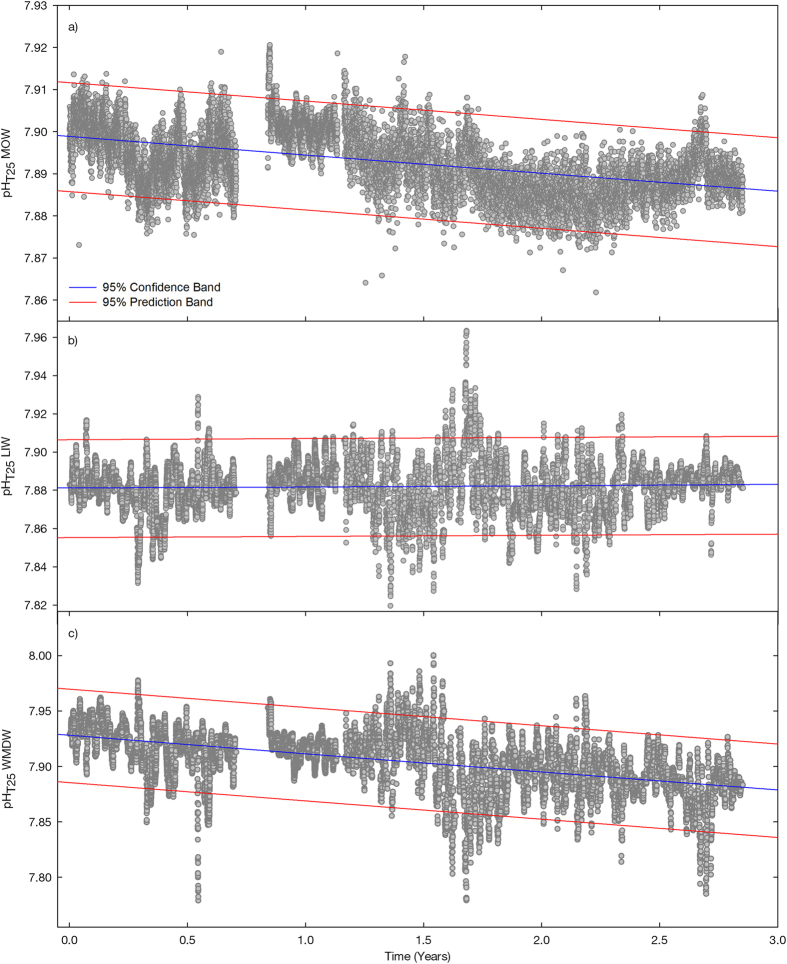
Linear fitting of pH with time (see SI *text*) of the MOW and its forming water masses during the monitoring period: (a) MOW, (b) LIW and (c) WMDW. Blue and red lines represent the 95% confidence and prediction bands, respectively. Equations are shown in the SI *text*. Note the different scales for “y” axes in figures (**a–c**).

As each water mass is characterized by a distinctive salinity and potential temperature, pH can also act as a tracer to define water masses. The OMP and MLR analysis allowed determining that LIW and WDMW in the SG were characterized by average pH_T25_ and standard error values of 7.8897 ± 0.0003 and 7.9077 ± 0.0004, respectively (see Fig. 4). Those values faithfully correspond to recently reported measures^18^ equal to ~7.89 for the LIW in the SG and 7.9–7.91 for the WMDW in the Western Mediterranean basin. Differences in pH values betwen both water masses can be explained on the basis of the transit times from their respective formation sites to the SG; the LIW takes around 8 years to complete the distance from the Levantine basin (LB, Fig. S1) to the Strait of Sicile (SS, Fig. S1)^32^. An active remineralization of organic matter can take place during such period, which implies the rise of dissolved inorganic carbon concentration, the decrease of dissolved oxygen and the consequent pH decrease^16^,^18^. In contrast, the WMDW takes roughly 1.8 years to travel from the Gulf of Lions to the Alboran Sea (GL and AS respectively in Fig. S1), a much shorter transit time^33^. Although the ventilation time of LIW and WMDW is an issue that has not been solved yet and there are still large discrepancies regarding the age of each water mass^34^,^35^,^36^,^37^, estimated ages are around 80–120 ± 20 yr and 20–40 ± 40 yr for the bottom waters of the eastern and western basins, respectively, when they arrive at the SG^35^. Therefore, considering the circulation pattern in the MS^38^ and regardless of a particular transit time or age for each water mass, when they both arrive at the SG the WMDW is much younger than the LIW formed in the distant Levantine Basin (LB, Fig. S1). Accordingly, the LIW is more stable than the WMDW from a biogechemical point of view, as the former lost contact with the atmosphere a longer ago. Therefore, in the SG, the WMDW,which has been exposed to the atmosphere more recently, exhibits higher pH values than the eastern-originated LIW (Fig. 4). This is confirmed when the monthly variability of pH in both water masses is analysed (see Fig. S2). Although a little seasonality can be detected, over the summer months, the two water masses showed stable pH values whereas winter conditions resulted in the highest pH variability, which was especially remarkable in February. During this month, pH in the WMDW presented noticeable oscillations, ranging from 7.7789 to 8.0003 whereas the pH of the LIW varied slightly, with values changing from 7.8195 to 7.9634. This seasonal variability could be again attributed to the ventilation pattern of the WMDW. A previous work^25^ demostrated that a lag of few weeks can be found between the events of WMDW fomation in winter in the Gulf of Lions and the detection of cold pulses of old WMDW at the monitoring site in Espartel Sill. Renovation of the old resident WMDW in the SG depends on the volume of water formed during winter by deep convection in the Gulf of Lions and the intensification of the WAG that subsequently uplifts the ancient WMDW at the SG. During our study period, the appearance of colder (and older) WDMW pulses in February 2013, 2014 and 2015 (Figs 1a and 2b) possibly resulted in the lowest pH values detected in the WMDW during the months of February (see Fig. S2b), as the older WMDW residing in the eastern side of the Strait will be characterized by lower pH values, due to the active remineralization processes occurring in the Alboran basin^39^.

### Trends of pH decrease in Mediterranean waters

The pH data series clearly depicted a negative trend (Fig. 1) although the data gap after the first period (April-June 2013) seems to visually break the tendency. From June 2013 onwards, the decreasing trend was even clearer. Taking into account the whole period, a pH-time linear regression was calculated (see *SI text* for calculation details) and plotted in Fig. 4. The regression statistics were significant and resulted in a ΔpH of −0.0044 units per year in the MOW. This rate of pH decline is of the same order of magnitude than acidification rates reported by sustained observations of different carbon system parameters collected in several oceanic seasurface time series^21^, although it is two or three fold higher than those (depending on the site). Nevertheless, it still falls within the range of pH change in Mediterranean deep waters estimated recently through a modelling approach (−0.005 to −0.06 pH units^13^).

The consistency of our pH decreasing pattern is supported by the complementary *p*CO_2_ measurements taken in the SG at the same sampling frequency. When the Δ*p*CO_2_ in the MOW obtained during the monitoring period is calculated, an increase in *p*CO_2_ of 5.1 μatm y^−1^ is obtained (see *SI text* and Fig. S4 for calculation details). This CO_2_ rise is coherent with (and matches) the rate of pH decline observed in the MOW.

Performing a separate analysis for each of the two main water masses forming the MOW, annual decline rates of −0.0006 and −0.0165 pH units per year are calculated for the LIW and WMDW, respectively (Fig. 4b,c). The regression descriptors were statistically significant (see *SI text*). These pH changes correspond to *p*CO_2_ variations of −0.6 and 18.5 μatm in each water mass respectively (see Fig. S4), according to the CO_2_ data in the SG.

The low ΔpH_LIW_ may be well attributable to its age (in the order of 100 years upon arrival at the SG) and stability, as this intermediate water mass has not been affected by the present atmopheric CO_2_ concentrations. On the other hand, the more recently produced WMDW showed a considerable pH decreasing trend and a noticeable *p*CO_2_ rise. As previously noted, this may be a consequence of several processes. First, the impact of higher atmospheric CO_2_ concentration this water mass was exposed to when formed, secondly, the faster penetration of CO_2_ in the water column brought about by the high alkalinity of this water mass^40^ and finally the sinking of labile and easily oxidizable organic matter facilitated by the formation events. Also, both LIW and WMDW reflect the gradient of trophic conditions from the Western to the Eastern Mediterranean basin. With a eastward decreasing gradient pattern of primary productivity^41^, the Alboran Sea represents one of the most productive Mediterranean areas^41^ involving a greater deep degradation potential of organic inputs coming from the photic zone^42^. Therefore, the WMDW, support high rates of organic carbon degradation^42^,^43^ and subsequently an increase on the CO_2_ content while residing in the deep Alboran Sea.

Observations gathered in other time series have shown that ocean regions characterized by high rates of increase in *p*CO_2_ (Irminger Sea and CARIACO, for instance) depict the highest rates of decrease in surface seawater pH (around −0.0025 pH units y^−1^)^21^.

Even though the time period of observations used in our work (roughly 3 years) can be considered relatively short to asses long term changes in the pH of the Mediterranean Sea, it still provides a reasonable wealth of data with sufficient sensitivity and accuracy to establish seasonal and interannual pH variations in the water masses of the Mediterranean Sea that reflect changes in both the natural carbon cycle and anthropogenic perturbation. It is worthy to point out that, in addition to the uptake of anthropogenic CO_2_ from the atmosphere, the Mediterranean basin receives continuously a considerable amount of anthropogenic carbon from the North Atlantic Ocean through the SG^20^. Monitoring the magnitude of the resulting pH decline in the basin requires sustained high-accuracy observations. The data presented here may well serve as a base line to assess sensitiviy and evolution of the Mediterranean waters to the impact of increasing CO_2_ emmissions.

## Methods

pH measurements shown in this study were collected from August 2012 to June 2015 at the GIFT time series in the Strait of Gibraltar (see *SI text* for mooring line details, Fig. S1). Water masses fractions within the MOW were obtained by an Optimum MultiParameter (OMP) analysis and modelled pH was constructed by performing a Multiple Linear Regression (MLR) least square fitting. In order to smooth out the short-scale variability and turbulence originated in the supercritical-to-subcritical flow transitions^44^ as well as the fluctuations associated with the tidally-generated short internal waves in the SG, which result mostly in the rapid mixing between water masses, data were averaged in periods of 84 hours (half week), as our work was meant to focus on long-term variability related to basin scale processes, which is relevant for OA signals. Details regarding the CO_2_ system calculations, data sets used and the statistical treatment may be found in SI (see *SI text*).

## Additional Information

**How to cite this article**: Flecha, S. *et al.* Trends of pH decrease in the Mediterranean Sea through high frequency observational data: indication of ocean acidification in the basin. *Sci. Rep.* **5**, 16770; doi: 10.1038/srep16770 (2015).

## Supplementary Material

Supplementary Information
