# Supplementary material for: Trends of pH decrease in the Mediterranean Sea through high frequency observational data: indication of ocean acidification in the basin
Source: Sci Rep. 2015 Nov 26;5:16770. doi: 10.1038/srep16770 (PMC4660394; doi:10.1038/srep16770)
Supplement: Supplementary Information [file srep16770-s1.pdf]

Supplementary Information (SI) of:

Trends of pH decrease in the Mediterranean Sea through high frequency observational data: indication of ocean acidification in the basin

Susana Flecha[1*], Fiz F. Pérez[2], Jesús García-Lafuente[3], Simone Sammartino[3], Aida. F. Ríos[2] and I. Emma Huertas[1*]

SI Text

Data recording. The mooring line deployed in the Espartel Sill (ES) consists of an uplooking Acoustic Doppler Current Profiler (ADCP) (Teledyne RD Instruments, Inc.), a currentmeter Nortek Aquadopp (Nortek AS), a CT (Conductivity Temperature) SBE 37 probe (Sea-Bird Electronics, Inc.), and SAMI-pH and SAMI-CO_2 Submersible Autonomous Moored Instruments (Sunburst Sensors, LLC.). CT, SAMI-pH and SAMI-CO_2 were placed around 13 m above the sea bottom (360 m depth) (Fig. S1). Precision and accuracy of measurements were < 0.001 and ± 0.003 pH units and < 1 ppm and ± 3 ppm for SAMI pH and pCO_2 instruments, respectively. Calibrations of instruments were performed by the manufacturer companies for the SAMI-CO_2 and for two CT's used periodically. SAMI-CO_2 used was acquired and calibrated previously to the deployment. CT's drifts obtained of +0.0001 and -0.00006 ºC/year and 0.0001 and 0.0000 PSU/month for temperature and salinity, respectively, were applied to the data. SAMI-pH data were validated with periodically laboratory calibrations by using Certified Reference Material (CRM supplied by Prof. Andrew Dickson, Scripps Institution of Oceanography, La Jolla, CA, USA) and a Shimadzu UV-2401PC spectrophotometer containing a 25 °C-thermostated cells holder

following a spectrophotometric method[1]. In addition, the drift obtained for the SAMI-pH from the manufacturer of 0.001pH over 6 months was applied to the obtained data.

pH data can be divided in 3 periods: 1) August 2012-April 2013, 2) June 2013-September 2013 and 3) October 2013-June 2015. During the first and third periods data were collected with the SAMI-pH sensor every 60 and 120 minutes, respectively. The sampling interval was changed in order to extend the batteries life and to ensure data acquisition during a longer period because, occasionally, the line cannot be recovered when planned due to adverse meteorological conditions in the Strait. In fact, data were missed during the second period because the batteries ran out before the devices could be lifted. To fill the data gap occurred in summer 2013, pH data were calculated from: the pCO_2 data obtained with the SAMI-CO_2 (which was operative during such period as the batteries had a longer life time), the Total Alkalinity (A_T)-Salinity relationship reported previously [2] in the area by using the carbonic acid apparent dissociation constants more appropriate for the SG waters [3,4].

CT data were recorded every 30 minutes and practical salinity (TEOS-10), salinity hereinafter, and potential temperature (Θ) were used afterwards to compute pH at *in situ* salinity and resolve the water masses structure within the MOW by an Optimum MultiParameter Analysis (OMP). Temperature and salinity chosen for OMP water masses characterization end members were 13.22, 12.8, 15º C and 38.56, 38.45, 36.8 for LIW, WMDW and AW respectively[5].

pH modeling, Trend Determinations and Statistics. pH was modeled through the classical MLR scheme that allows establishing the relationship between a response variable (pH) and some predictors (in this case the fraction of the three water masses that can be found in the Strait, fAW, fWMDW and fLIW) by using a linear combination of the predictors. The result of our data fitting was:

$$pH \pm 0.0073 = 7.8577 \pm 0.0018 * fAW + 7.9077 \pm 0.0004 * fWMDW + 7.8897 \pm 0.0003 * fLIW$$

(R^2=0.999; n=15937),

where the predicted variable, the obtained coefficients and the standard error are indicated. Comparable results were obtained with pCO_2 as the predicted variable (Fig. S3).

Trends in pH and pCO_2 were determined by ordinary linear regression and standard errors and 95% confidence intervals were calculated for the slopes of the regressions. In the case of the MOW, pH linear fitting with time is represented by the equation:

$$pH_{MOW} = -0.0044 \pm 6.2094 * 10^{-5}\ t + 7.8987 \pm 9.1824 * 10^{-5}$$ ($R^2 = 0.2362$; $p<0.01$; n=15937),

whereas the results of the fitting for the WMDW and LIW were given by the following expressions:

$$pH_{WMDW} = -0.0165 \pm 0.0002\ t + 7.9276 \pm 0.0003$$ ($R^2 = 0.2946$; $p<0.01$; n=15937),

$$pH_{LIW} = -0.0006 \pm 0.0001\ t + 7.8822 \pm 0.0002$$ ($R^2 = 0.0011$; $p<0.01$; n=15937),

where t represents time (years) starting from August 8th , 2012 to June 15th ,2015. Standard errors are also shown.

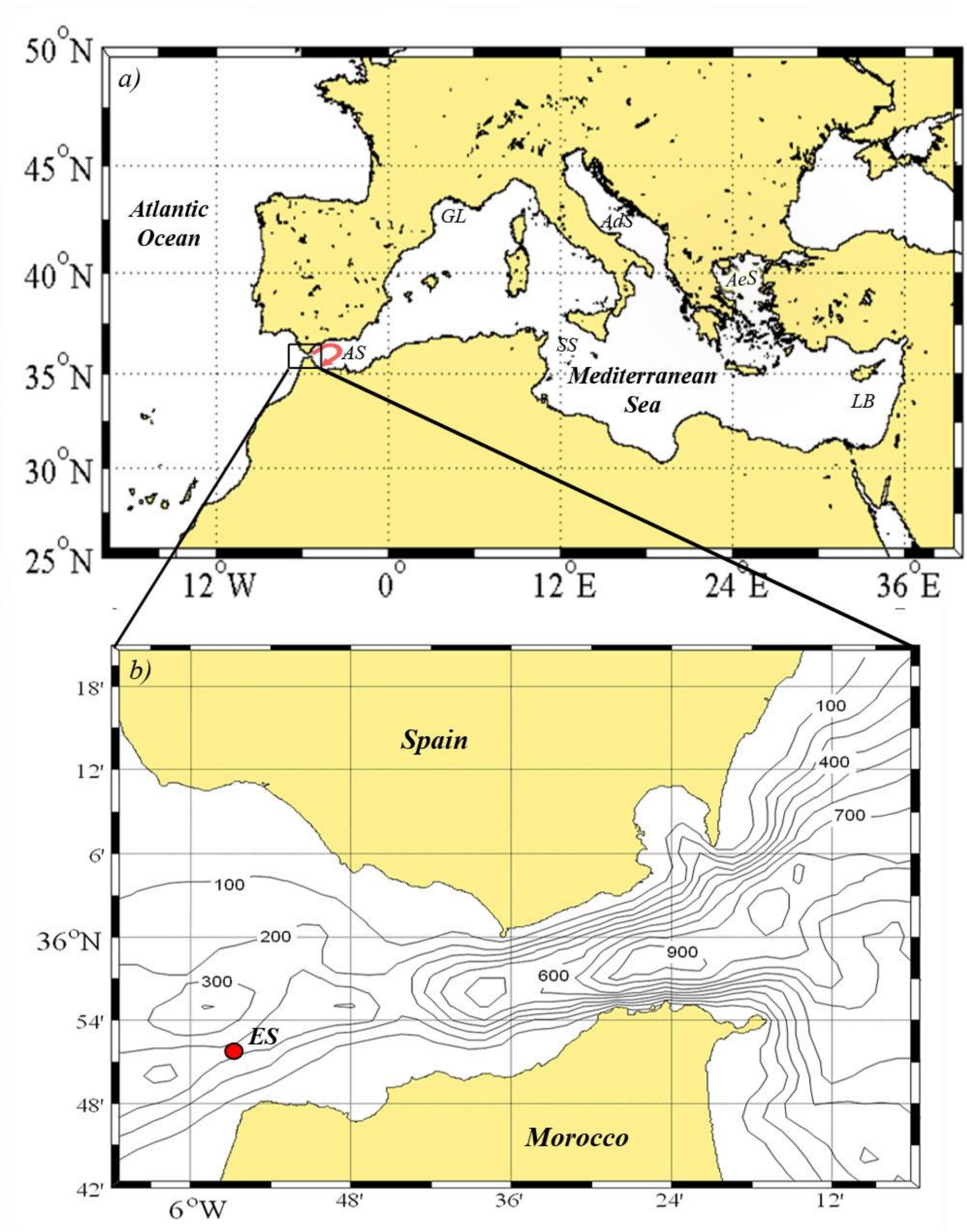

Fig. S1: a) Map of the Mediterranean Sea, detailing the location of different sub-basins: Alboran Sea (AS), Gulf of Lions (GL) Adriatic Sea (AdS), Aegean Sea (AeS), Levantine Basin (LB) and the Strait of Sicily (SS), b) map of the Strait of Gibraltar showing the mooring line location at the Espartel Sill (ES). Maps were developed with the MATLAB® software by using the M_Map toolbox.

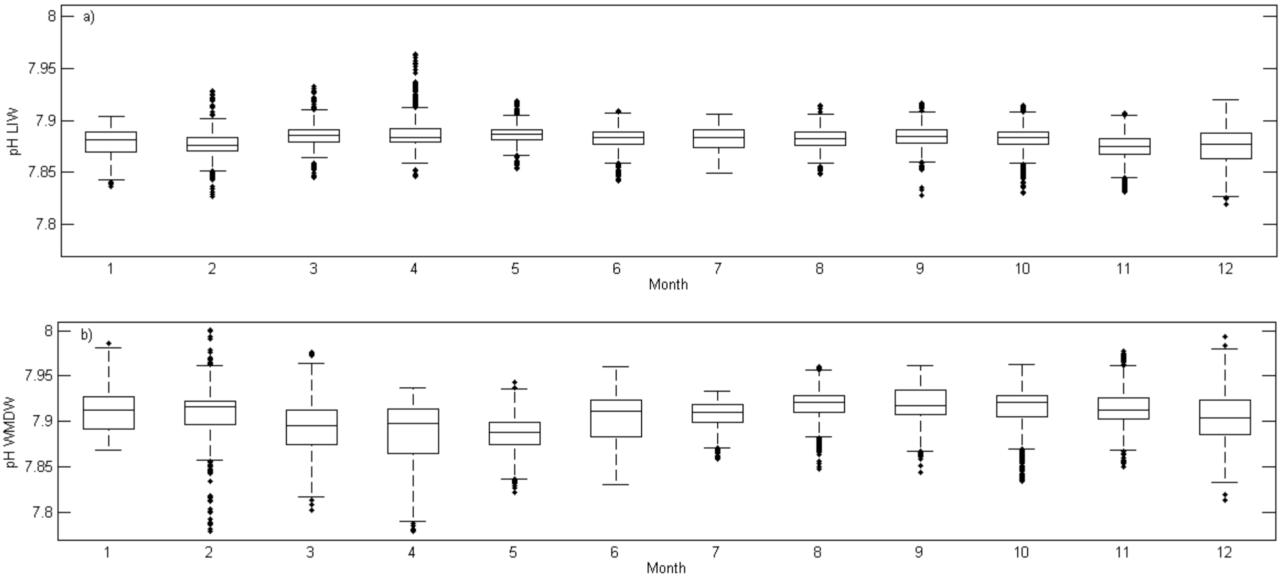

Fig. S2: Monthly pH values of the water masses forming the MOW during the monitoring period: a) LIW and b) WDMW. The tops and bottoms of each “box” are the 25th and 75th percentiles of the samples, respectively. The distances between the tops and bottoms are the interquartile ranges whereas the line in the middle of each box is the sample median. Observations beyond the whisker length are marked as outliers displayed with a black dot.

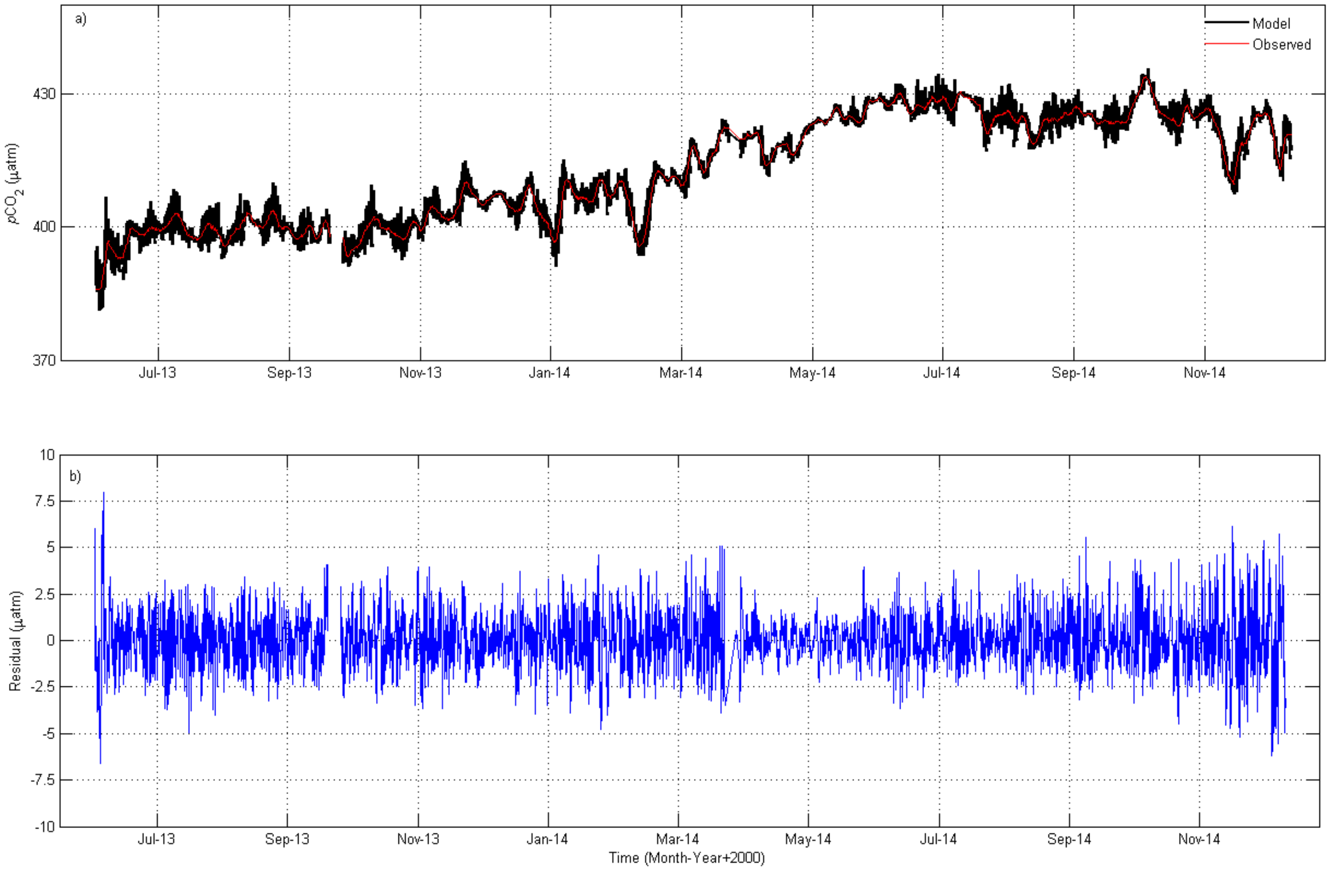

Fig. S3: a) pCO_2 obtained with the SAMI device averaged to 84 h (red line) between June 2013 and November 2014 and modelled pCO_2 (black line), b) Residuals between observed values and modelled outputs.

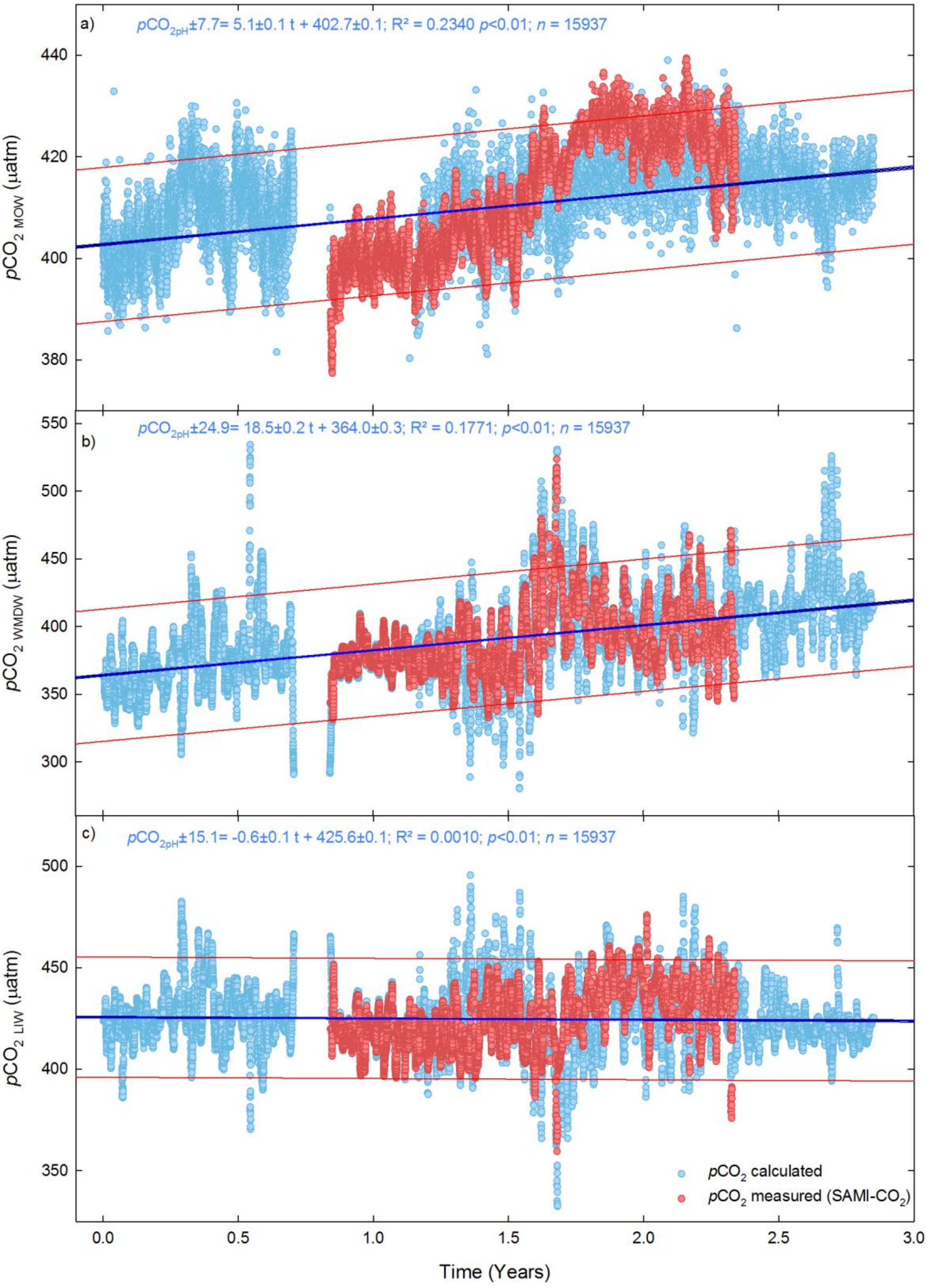

Fig. S4: Linear fitting of pCO_2 with time (see SI *text*) of the MOW and its forming water masses during the monitoring period: a) MOW, b) LIW and c) WMDW. Blue and red lines

represent the 95% confidence and prediction bands, respectively. Note the different scales for “y” axes in figures a, b and c.

References

1. Clayton TD, Byrne RH. Spectrophotometric seawater pH measurements: total hydrogen ion concentration scale calibration of m-cresol purple and at-sea results. *Deep Sea Research Part I: Oceanographic Research Papers* **40**, 2115-2129 (1993).

2. Huertas IE, *et al.* Anthropogenic and natural CO_2 exchange through the Strait of Gibraltar. *Biogeosciences* **6**, 647-662 (2009).

3. Mehrbach C, Culberson CH, Hawley JE, Pytkowicz RM. Measurement of the apparent dissociation constants of carbonic acid in seawater at atmospheric pressure. *Limnology and Oceanography* **18**, 897-907 (1973).

4. Dickson A, Millero F. A comparison of the equilibrium constants for the dissociation of carbonic acid in seawater media. *Deep Sea Research Part A Oceanographic Research Papers* **34**, 1733-1743 (1987).

5. García Lafuente J, Sánchez Román A, Díaz del Río G, Sannino G, Sánchez Garrido J. Recent observations of seasonal variability of the Mediterranean outflow in the Strait of Gibraltar. *Journal of Geophysical Research: Oceans (1978–2012)* **112**, C10005, (2007). doi:10.1029/2006JC003992.
